# Superinfection occurs in *Anaplasma phagocytophilum *infected sheep irrespective of infection phase and protection status

**DOI:** 10.1186/1751-0147-51-41

**Published:** 2009-10-26

**Authors:** Snorre Stuen, Wenche O Torsteinbø, Karin Bergström, Kjetil Bårdsen

**Affiliations:** 1 Norwegian School of Veterinary Science, Department of Production Animal Clinical Sciences, Sandnes, Norway; 2 National Veterinary Institute, Department of Bacteriology, Uppsala, Sweden

## Abstract

**Background:**

*Anaplasma phagocytophilum *infection in domestic ruminants is widespread in the coastal areas of southern Norway. The bacteria may persist in mammalian hosts. Several genetic variants of *A. phagocytophilum *exist. In the present study, we investigate whether superinfection occurs in the acute and persistent phase of the infection.

**Methods:**

Five-month-old lambs of the Norwegian Dala breed were experimentally infected with two 16S rRNA gene variants of *A. phagocytophilum*, i.e. *A. phagocytophilum *variant 1 (GenBank accession number M73220) and variant 2 (GenBank acc. no. AF336220). Eighteen lambs were used, two lambs in each group. Eight groups were experimentally inoculated with either variant 1 or 2 on day 0. Six of these groups were then challenged with the other variant on either days 7, 42 or 84, respectively. One group was left uninfected. The occurrence of *A. phagocytophilum *in blood samples was determined using semi-nested PCR analysis and gene sequencing. Specific antibodies were measured by an indirect immunofluorescence antibody assay (IFA).

**Results:**

*A. phagocytophilum *variant 1 and 2 differed significantly with regards to clinical reaction and cross-immunity in infected lambs. Both variants were found in the blood after challenge. However, variant 1 was detected most frequently.

**Conclusion:**

The present experiment indicates that superinfection of different genotypes occurs during the acute as well as the persistent phase of an *A. phagocytophilum *infection, even in lambs protected against the challenged infection.

## Background

The rickettsia *Anaplasma phagocytophilum *(formerly *Ehrlichia phagocytophila*) causes tick-borne fever (TBF) in domestic ruminants. The disease has also been diagnosed in several other animal species and in humans [1-3]. In Europe, *A. phagocytophilum *is mainly transmitted by the tick *Ixodes ricinus*. The infection has for decades been one of the main scourges for the Norwegian sheep industry [4]. A serological survey in sheep indicated that *A. phagocytophilum *infection is widespread along the coast of southern Norway [5].

Based on 16S rRNA and *msp4 *gene sequence studies, several variants of *A. phagocytophilum *exist simultaneously in the same sheep flock [6]. These variants may cause different clinical manifestations [4]. Previously it has been proposed that unidirectional suppression of genotypes occurs in lambs infected simultaneously with different variants and that variants may cycle differently in the mammalian host [7,8].

Superinfection, i.e. establishing of a second variant of a strain in a host already infected with a primary variant, has been demonstrated in the closely related organism, *A. marginale *[9,10]. In the present study, we investigate whether superinfection occurs in *A. phagocytophilum *infected lambs by using two 16S rRNA gene variants of the bacterium.

## Methods

### Source of A. phagocytophilum

Blood samples were originally collected from a flock of Norwegian Dala sheep infected with *A. phagocytophilum*. EDTA and heparinised blood samples were collected from infected lambs. Two different 16S rRNA gene variants, i.e. *A. phagocytophilum *variant 1 (GenBank accession number M73220) and variant 2 (GenBank acc. no AF336220) were obtained from two lambs, each infected with one of the variants [11]. Both variants have earlier been used in several inoculation studies without indication of a mixed infection in the original blood material [8,12].

The EDTA blood samples from the original infected lambs were used to measure haematological values and to prepare blood smears. The absolute number of infected cells per unit volume was determined by multiplying the total number of neutrophils per unit volume by the percentage of infected neutrophils counted on a May-Grünwald Giemsa stained blood smear. The heparinised blood samples were stored at -70°C with 10% dimethyl sulphoxide (DMSO) as cryoprotectant without any propagation in cell culture or sequence passages through other sheep.

### Animals, experimental design, and haematology

Eighteen 5-months-old lambs of the Dala breed were used in this trial. The lambs were unrelated and belonged to the experimental sheep flock at the Department of Production Animal Clinical Sciences. The experiment was approved by the National Animal Research Authority (Norway). None of the lambs had previously been on *I. ricinus*-infested pasture and were kept indoors during the whole experimental period of four months. In addition, prior to the first inoculation, the lambs were tested for an *A. phagocytophilum *and a *Mycoplasma *(formerly *Eperthrythrozoon*) *ovis *infection by blood smear examinations. Nine groups each with two lambs were formed by random sampling. Four groups were inoculated intravenously with 1 ml of a whole blood DMSO-stabilate of *A. phagocytophilum *variant 1 and four other groups were inoculated with 1 ml of a stabilate of *A. phagocytophilum *variant 2 (day 0). Six inoculated groups were then challenged with the different variant on either days 7, 42 or 84, respectively. The infectious blood of both variants contained approximately 0.5 × 10^6 ^infected neutrophils/ml. One group was left uninfected as controls.

Rectal temperatures were recorded daily in all lambs throughout the experimental period. The incubation period was defined as the period between inoculation and the first day of fever (≥40.0°C). Other clinical variables such as fever response and duration of neutropenia (<0.7 × 10^9 ^cells/l) were recorded as described by Stuen et al. [13].

Blood samples were collected daily into EDTA-coated tubes during the febrile period following inoculation of the infected blood, and then every second day after the fever had subsided. From these blood samples haematological values including total and differential leucocyte counts were determined electronically (ADVIA, Bayer).

### DNA amplification and sequence analysis

DNA amplifications were carried out on a PTC-200 instrument (MJ Research) as previously described [8]. Briefly, an initial PCR was performed using primers 16S-F5 (5'-AGTTTGATCATGGTTCAGA-3') and ANA-R4B (5'-CGAACAACGCTTGC-3') for amplification of a 507 bp fragment of the 16S rRNA gene in *A. phagocytophilum*. The subsequent semi-nested reaction with primers 16S-F5 and ANA-R5 (5'-TCCTCTCAGACCAGCTATA-3') produced a 282 bp fragment. PCR products were sequenced directly using Big Dye terminator cycle sequencing chemistry and capillary electrophoresis (ABI 310; Applied Biosystems) and *A. phagocytophilum *variants were detected from visual inspection of the sequence data [8].

### Serology

Sera were collected at days 0, 7, 14, 21, 28, 35, and 42 after each inoculation and analysed for antibodies against *A. phagocytophilum *using an indirect immunofluorescence antibody assay (IFA) [5]. Briefly, two-fold dilutions of sera were added to slides precoated with *A. phagocytophilum *(formerly *Ehrlichia equi*) antigen (Protatec, St. Paul, Minn.). Bound antibodies were visualized by fluorescein-isothiocyanate (FITC)-conjugated rabbit-anti-sheep immunoglobulin (Cappel, Organon Teknika, West Chester, PA). Sera were screened for antibodies at dilution 1:40. If positive, the serum was further diluted and retested. A titer of 1.6 (log10 reciprocal of 1:40) or more was regarded as positive.

### Statistics

Statistical analysis was performed using a two-sample *t-*test (Statistix^®^, version 4.0; Analytical software). A *P *value of < 0.05 was considered significant.

## Results

### Clinical parameters and haematology

All lambs primarily infected with *A. phagocytophilum *developed fever and neutropenia. Differences in clinical signs and hematological reactions were observed after the primary infection (Table 1). In addition, lambs infected with variant 1 showed no clinical or haematological reactions when challenged with variant 2 on days 7 and 42, and only one day with mild fever (T<41.0°C) was observed when challenged on day 84. However, lambs primary infected with variant 2 reacted with fever (one day, T<41.0°C) and neutropenia when challenged with variant 1 on day 7, being fully susceptible to *A. phagocytophilum *var 1 infection when challenged on day 42 and 84 (data not shown). No clinical reactions were observed in the controls.

**Table 1:** Clinical manifestations and immunofluorescent antibody titre (mean ± std) in lambs primary infected on day 0 with either *A. phagocytophilum *var 1 or var 2

	***A. phagocytophilum var 1 (n = 6)***	***A. phagocytophilum var 2 (n = 6)***
Incubation period (days)^#^	3.7 ± 0.47	4.5 ± 0.50

Max. temp. (°C)^##^	41.6 ± 0.15	40.9 ± 0.33

Duration of fever (days)^###^	6.0 ± 0.82	3.0 ± 0.58

Nadir of neutropenia (G/l)^###^	0.3 ± 0.08	0.6 ± 0.11

Duration of neutropenia (days)^###^	8.6 ± 1.10	2.3 ± 0.75

Antibody titre (log_10_), day 14^###^	3.4 ± 0.11	2.3 ± 0.15

Antibody titre (log_10_), day 28^###^	3.3 ± 0.37	2.3 ± 0.24

^# ^P < 0.05, ^## ^P < 0.01, ^### ^P < 0.001

### 16S rRNA gene sequencing

Both variants of *A. phagocytophilum *were detected after reinfection. After challenge with variant 2 on day 7, variant 1 was detected 13 times, while variant 2 was detected at 5 occasions. In addition, when challenged on day 7 with variant 1, variant 1 and 2 was detected 12 and 6 times, respectively. Similar results were obtained when lambs were challenged on day 42 and day 84, respectively (Table 2). Infection was not detected in the controls.

**Table 2:** The occurrence of *A. phagocytophilum *variant 1 and 2 in the peripheral blood of experimentally inoculated lambs

***Days after challenge***	***Lambs a/b***	***Lambs c/d***	***Lambs e/f***	***Lambs g/h***	***Lambs i/j***	***Lambs k/l***
0*	1/1	1/-	-/-	2/2	2/2	2/-
3	1/1	1/2	2/2	2/2	1/1	1/1
5	1/1	2/2	2/2	1/1	1/1	1/1
7	-/-	2/-	2/2	1/1	1/1	1/1
10	-/1	2/1	1/-	1/1	1/1	1/1
12	1/1	-/-	-/-	-/-	1/1	1/1
14	1/-	2/2	1/2	1/2	-/1	-/2
17	2/-	-/2	-/-	-/1	-/1	1/2
21	2/-	-/-	-/-	-/1	-/2	-/1
24	2/2	1/1	-/-	2/2	2/2	-/-
28	2/-	1/1	-/1	-/2	-/-	-/-
31	-/1	-/-	1/1	-/-	1/-	1/-
35	1/-	1/-	1/-	-/-	-/-	-/1
38	1/1	-/-	-/-	1/-	1/1	1/-
42	1/-	-/1	-/-	1/1	1/-	1/1

Two lambs in each group. Lambs a and b; c and d; e and f were infected with variant 1 on day 0 and challenged with variant 2 on day 7, 42 and 84, respectively. Lambs g and h; i and j; k and l were infected with variant 2 on day 0 and challenged with variant 1 on day 7, 42, and 84, respectively.

1 - variant 1, 2 - variant 2, - no detection, * infection prior to challenge

### Serology

**Figure 1:**
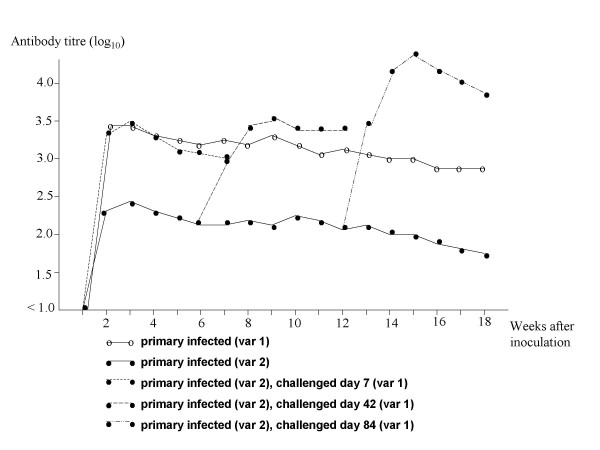
**Mean immunofluorescent antibody titre to *A. phagocytophilum *in lambs experimentally infected with two variants (variant 1 and 2) at different time points**. Two lambs in each group. A titre below 1:40 (log_10 _= 1.6) was considered negative.

The primarily infected lambs developed specific antibodies within two weeks after inoculation. Lambs infected with variant 1 responded with the highest antibody titre (Table 1). Lambs primary infected with variant 2, showed an increase in antibody titre after challenge with variant 1 on days 7, 42 and 84, respectively (Fig. 1). In contrast, challenge with variant 2 did not affect the antibody titre in lambs already infected with variant 1 (data not shown). The controls were seronegative.

## Discussion

Following the primary inoculation, all lambs responded with typical signs of *A. phagocytophilum *infection [13]. Although a small number of lambs were used, the present study indicates that lambs infected with variant 1 revealed a more severe clinical response compared with lambs infected with variant 2. Also after challenge there is a marked difference in clinical reaction in lambs previously infected with variant 1 compared with variant 2. The results are in accordance with earlier experimental studies, where lambs infected with variant 1 had a more severe clinical manifestation compared with lambs infected with variant 2 [12].

After challenge, variant 1 was detected more frequently compared with variant 2. Variant 1 and 2 differ by a single polymorphism making identification and separation of their respective PCR products difficult. Therefore, PCR products were sequenced for the precise detection of the variants. However, due to variation in expression levels in variant 1 and 2, sequencing might not have identified all positive blood samples, at least samples containing a mixed infection. To ensure the detection of both variants a designed plasmid containing the mutation in the 16S rRNA gene should have been constructed from all samples. Unfortunately, this method was not available. However, in accordance with earlier studies, the present result indicates that variant 1 cycles more frequently than variant 2 in persistently infected lambs [7]. In addition, previous studies indicate that variant 1 is involved in most fatal cases of TBF and seems to be the most widespread variant in sheep in Norway [14]. The reasons for this dominance are unknown, but factors such as growth rate, immunogenicity, receptor competition, cyclic variation and antigenic variation may be involved in the infectivity and interaction of *A. phagocytophilum *variants in natural hosts [15-17]. Futhermore, different intrinsic transmission efficiency in *I. ricinus *ticks between variants of *A. phagocytophilum *may occur, as has been shown for strains of *A. marginale *in the tick *Dermacentor andersoni *[17].

The infected lambs developed a positive antibody titre to *A. phagocytophilum *between 7 and 14 days post inoculation with a marked difference in the antibody response between the two strains. Strong serological cross-reactions between all members of the *A. phagocytophilum *group have been reported, but the antibody titre to a heterologous variant is normally less that that to a homologous variant [18]. Unfortunately, the *E. equi *antigen was the only antigen that was available for use in the present study. However, a marked difference in the serological response has also earlier been observed after experimental infection with the same two variants of *A. phagocytophilum *[12].

Lambs primary infected with variant 1 did not seroreact after challenge with variant 2, in contrast to lambs challenged with variant 1. Lambs infected with variant 1 seem to be fully protected against variant 2 infection both in the acute and the early persistent phase, but not vice versa. Although few lambs were used in this study, the result is in accordance with earlier crossprotection studies involving the same variants of *A. phagocytophilum *[12].

The present study indicates that lambs infected with one variant of *A. phagocytophilum *may become infected with another variant both in the acute and the persistent phase of the infection. In addition, superinfection may occur without resulting in any clinical signs in the host and in lambs apparently fully protected against reinfection. The results indicate that superinfection occurs in any stage of the infection and seems to be independent of the variant involved.

In the present study, only needle inoculation was used. Earlier studies have shown that the tick itself can modulate the host's immune response [19]. To examine the present results, similar studies with the natural vector should be performed. However, marked differences in clinical symptoms and course of the infection following infection from either ticks or from inoculation by needle, have not earlier been recorded in sheep, horses and mice [20-22].

The epidemiological implications of superinfection are unknown. The prevalence of *A. phagocytophilum *infection in *I. ricinus *ticks in Europe has been found to vary from 1.5% to 34% [23,24]. Natural hosts may therefore be infested with several *A. phagocytophilum *infected ticks either simultaneously or during the grazing season. Simultaneous infection with two variants of the bacterium has earlier been observed in both cattle and sheep [11,14]. If superinfection occurs frequently, this indicates that naturally hosts may harbour an increasing number of variants during the grazing season. This statement is supported by a field study, where an increasing number of *A. phagocytophilum *variants were obtained in the blood of infected lambs throughout a three-month grazing period [6]. At the end of that study, 12 of 16 lambs (75%) were found infected with more than three *msp4 *gene variants, which indicates that superinfection may occur even in lambs already infected with several variants of the bacterium. Mixed infection of *A. phagocytophilum *in natural hosts should therefore be suspected under field conditions. Earlier observations indicate that mixed infection of *Borrelia burgdorferi *species in the natural host *Peromyscus leucopus *is more the rule than the exception [25]. However, due to limitation in sensitivity in the detection method, infection suppression, and cycling of variants in the mammalian host, it may be difficult to obtain all variants involved in naturally infected mammals, especially when analysing a limited number of blood samples [6-8].

## Conclusion

The present experiment indicates that superinfection of different genotypes occurs both during the acute and the persistent phase of an *A. phagocytophilum *infection, even in lambs protected against the challenged infection. Superinfection may play an important role in the maintenance of bacterial strains in the mammalian hosts [25]. Further studies are needed to address the implication of superinfection on the epidemiology of genotypes of *A. phagocytophilum *in several potential hosts and ticks, for instance to investigate if ticks could obtain and transmit several variants simultaneously and if genotypes are linked to specific mammalian hosts.

## Competing interests

The authors declare that they have no competing interests.

## Authors' contributions

SS has designed and performed the experimental study. SS carried out the statistical analysis and drafted the manuscript. WOT and KBA carried out the molecular genetic analyses. KBE performed the serology. All authors read and approved the final manuscript.
